# Genetic Counseling in Aotearoa New Zealand: Context, Practice and the Future of Genomic Healthcare

**DOI:** 10.1002/jgc4.70269

**Published:** 2026-07-17

**Authors:** Elisha Swainson, Harry G. Fraser, Linda L. Cheng, Sarah Collis, Alice Christian, Kimberley Gamet, Alison McEwen

**Affiliations:** ^1^ Genetic Health Service New Zealand, Te Whatu Ora Auckland New Zealand; ^2^ Graduate School of Health University of Technology Sydney Ultimo New South Wales Australia

**Keywords:** Aotearoa, genetic counseling, genetic counselors, genomics, Māori, New Zealand, workforce

## Abstract

Genetic counselors have been practicing in Aotearoa New Zealand since 1995, and are now established across both public and private sectors of healthcare. While significant global efforts have been made to define the profession of genetic counseling, the distinct way in which genetic counselors practice in New Zealand has not been formally described in the literature. The genetic counseling profession has evolved within New Zealand's distinct cultural, political, geographical, and healthcare ecosystem and is committed to addressing health inequities experienced by Māori, the Indigenous people of Aotearoa. Clinical practice is informed by *Te Tiriti o Waitangi* (the Treaty of Waitangi), and incorporates *te ao Māori* (Māori worldview), Māori ethical frameworks and Māori models of health. Genetic counselors work with Māori *whānau* (families) to facilitate collective decision‐making through *hui* (family meetings) for conditions such as familial hypercholesterolemia (FH), and contribute to Māori‐led research initiatives, including work leading to the discovery of the *CDH1* tumor suppressor gene. However, the genetic counseling workforce in New Zealand faces significant challenges, including limited local training opportunities, ongoing workforce constraints amid increased demand, the need to adapt to new models of service delivery, and underrepresentation of Māori and Pacific Peoples. This article provides the first published account of New Zealand's unique genetic counseling landscape and begins to map some of the strengths and challenges shaping the ongoing evolution of genetic counseling practice.

## Abstract (highlights)

**What Is Known About This Topic:**

Genetic counseling is an established profession internationally, and models of practice have been recognized and formally described in several countries. In Aotearoa New Zealand, Māori health frameworks and models of care are widely documented; however, the landscape of the genetic counseling profession has not been described in the literature.

**What This Paper Adds to the Topic:**

By providing the first published synopsis of a culturally shaped model of genetic counseling in Aotearoa New Zealand, this paper contributes to the international discourse on genetic counseling and demonstrates how locally responsive practice can inform global professional development.

## 1 Introduction

Genetic counseling has transitioned from a small, concentrated profession, established in the United States (US) in 1967 (Paneque et al. 2024), to one with a substantial and growing international presence. As of 2024, estimates indicate that more than 10,000 genetic counselors are practicing across 45 countries (Ormond et al. 2024), with approximately 450 employed clinically in Australasia. Although core ethical and medical principles are shared internationally (Rantanen et al. 2008), genetic counseling practice has diversified considerably across regions (Abacan et al. 2019; Ormond et al. 2018). Service delivery is ultimately shaped by local political, socioeconomic, and cultural factors, emphasizing that effective genetic counseling ought to be responsive to the needs of the communities it serves. Historically, dominant models of genetic counseling have been influenced by practice in the US and the United Kingdom (UK), reflecting the origins of the profession. However, implementation in diverse contexts requires careful alignment with the unique values and needs of local populations. This article describes how genetic counseling practice can be adapted to and embedded within existing Indigenous healthcare frameworks, ensuring the culturally sensitive provision of genetic counseling services while also contributing to the broader development of the genetic counseling profession.

## 2 The Aotearoa New Zealand Context

Aotearoa New Zealand (hereon New Zealand) has a well‐established public and private genetic counseling ecosystem, with the first genetic counselor employed in the public health service in 1995, and the national *Genetic Health Service New Zealand* (GHSNZ) established in 2012. Honoring *Te Tiriti o Waitangi* (New Zealand's founding document) and the needs of Māori (New Zealand's Indigenous people) is of central importance to genetic counseling theory and practice nationwide.

New Zealand is made up of two main islands and several smaller islands. Geographical access to genetic services is a challenge due to the nation's relatively small and sparse population. For example, New Zealand and the UK are comparable in terms of total land area, with New Zealand covering 268,021 km^2^ and the UK 242,495 km^2^. However, New Zealand's population of 5.34 million (The World Bank 2024) is overseen by three regional genetics services, while the UK's considerably larger population of 69.23 million (The World Bank 2024) is spread across 24 regional genetics services. In New Zealand, each service covers an average area of 89,340 km^2^, while those in the UK cover an area of only 10,104 km^2^.

Most healthcare services in New Zealand are government‐funded and administered by Health New Zealand, with private options also available. All New Zealand citizens, residents, and some temporary class visa holders are eligible for publicly funded healthcare (Health New Zealand 2025). Te Tiriti, signed in 1840 between Māori *rangatira* (chiefs) and the British Crown, declares Māori as *tangata whenua* (Indigenous peoples) and asserts ongoing rights to partnership, protection, self‐determination, and equity. As of 2025, an estimated 17.5% of the national population identified as Māori (Stats NZ 2025). Under Te Tiriti, the Crown is obligated to protect the health of Māori people and ensure equitable health outcomes (Durie 2004), a commitment reflected in New Zealand's Māori Health Strategy, *He Korowai Oranga* (Ministry of Health 2018b). However, Māori have experienced significant health disadvantages compared to non‐Māori since the 19th century, following European settlement and colonization (Durie 2004). A complex and longstanding interplay of socioeconomic and political factors has contributed to these health disparities (Ministry of Health 2018a).

## 3 Embedding *Kaupapa Māori* in Genetic Counseling Practice

Genetic counseling practice in Aotearoa is distinctly shaped by *te ao Māori* (the Māori worldview) and Māori ethical frameworks, including *He Tangata Kei Tua* (Hudson et al. 2016a) and *Te Mata Ira* (Hudson et al. 2016b). The He Tangata Kei Tua guideline describes a *tikanga Māori* (Māori protocols and practices) biobanking approach which recognizes Māori tissue, DNA, and subsequent data as *taonga* (precious treasure), imbued with *mauri* (the life force in people and objects), and therefore requiring *kaitiakitanga* (stewardship/Māori governance). *Whakapapa* (genealogy; ancestral link) is embodied in a person's DNA, making the storage and use of human tissue for genetic testing a process that carries significant spiritual meaning, necessitating culturally informed consent with the option of sample return or disposal (Hudson et al. 2016a). For example, during consent for genetic testing, whānau can request a *karakia* (prayer) be performed at sample disposal, acknowledging its *tapu* (sacred; spiritual) nature and releasing the *wairua* (spirit) of the sample (Beaton et al. 2017). Return of sample to the whānau post‐test is routinely offered, restoring the taonga back to the whānau. A glossary of te reo Māori terms used throughout this manuscript is provided in Table 1.

**TABLE 1:** Glossary of Te Reo Māori terms used throughout the manuscript.

Term	Definition
*He Tangata Kei Tua*	Māori ethical framework
*Hā a koro mā, a kui mā*	Inherited ancestral strengths
*He Korowai Oranga*	Māori Health Strategy of Aotearoa New Zealand
*Hinengaro*	Mind; thoughts; mental and emotional wellbeing
*Hui*	Meeting; gathering
*Iwi*	Tribe
*Kaitiakitanga*	Guardianship; stewardship
*Karakia*	Prayer; incantation
*Kaumātua*	Respected elders
*Kaupapa Māori*	Māori approach or framework
*Mana*	Authority; status
*Marae*	Communal meeting place
*Mauri*	Life force; vital essence
*Mihi*	Formal greeting or introduction
*Māori*	Indigenous peoples of Aotearoa New Zealand
*Meihana Model*	Māori model of health
*Pōwhiri*	Formal welcome ceremony
*Pūtahi Manawa*	“Healthy Hearts”, Māori cardiovascular research project
*Rangatira*	Chief; leader
*Rangatiratanga*	Self‐determination
*Tangata whenua*	People of the land; Indigenous peoples
*Taonga*	Treasured possessions; culturally significant resources
*Tapu*	Sacred
*Taha Hinengaro*	Mental and emotional health
*Taha tinana*	Physical health
*Taha Wairua*	Spiritual health
*Taha Whānau*	Family and social health
*Te ao Māori*	Māori worldview
*Te Mana Raraunga*	Māori Data Sovereignty Network
*Te Mata Ira*	Māori ethical framework
*Te Tiriti o Waitangi*	Treaty of Waitangi
*Te Whare Tapa Whā*	Māori model of health
*Te Wheke*	Māori model of health
*Tikanga Māori*	Māori customs, values, and practices
*Tinana*	Body; physical self
*Wairua*	Spirit
*Waiata*	Song
*Whakapapa*	Genealogy; ancestral connections
*Whānau*	Family; extended family network
*Wharenui*	Meeting house

**FIGURE 1:**
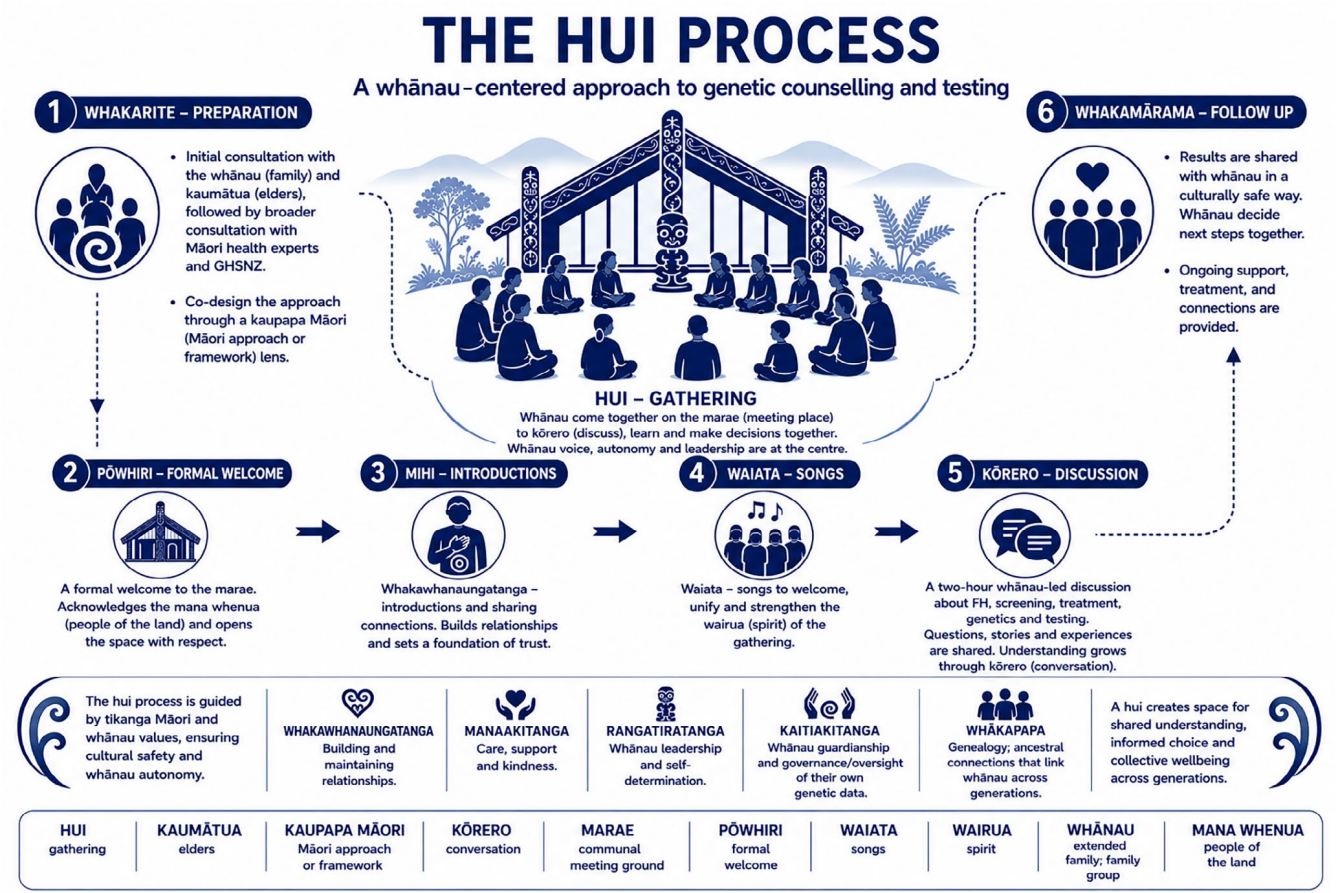
The Hui Process. This figure illustrates The Hui Process and key stages of engagement as applied to whānau‐centered genetic counseling within a tikanga Māori framework. This is a visual representation of the process described in “Hui: A partnership in practice in familial hypercholesterolemia” by J. Benatar et al. 2020, *The New Zealand Medical Journal*. Image designed using Claude 4.6 Sonnet, Anthropic 2026, 14 May 2026.

The Te Mata Ira framework describes a relational model of care, built on trust, reciprocity, and sustained partnerships with whānau and *iwi* (tribes). It recognizes whakapapa as both scientific and spiritual knowledge, and seeks to ensure that consent, data use, and communication uphold *man*a (status), tapu, and *rangatiratanga* (self‐determination) (Hudson et al. 2016b). Drawing on this framework, genetic counseling practice facilitates shared decision‐making within whānau groups and acknowledges collective rights to information obtained from whakapapa. These principles inform culturally aligned models of practice, such as *The Hui Process*, developed by Lacey et al. (2011). Genetic counseling is often offered to Māori whānau in the form of a *hui* (gathering), where family members are seen and, if appropriate, offered testing together. Adopting this model, researchers held a hui on the *marae* (meeting ground) in a remote region of Aotearoa, to offer cascade testing for familial hypercholesterolemia (FH) within a large extended Māori whānau (Benatar et al. 2020). This process (Figure 1) involved initial consultation with the whānau, *kaumātua* (elders), and Māori health experts to co‐design the testing approach through a *kaupapa Māori* (Māori approach or framework) lens. The hui was attended by a multidisciplinary team, including genetic counselors, and began with a *pōwhiri* (formal welcome), *mihi* (introductions) and *waiata* (songs), followed by a two‐hour discussion about FH, screening, treatment, genetics, and testing. A hui creates space for shared understanding to develop through whānau‐led discussion and culturally appropriate support. Conversations about whakapapa, genetic risk and testing are facilitated within a framework that respects tikanga Māori and whānau autonomy (Lacey et al. 2011).

The relatively small and often interconnected nature of New Zealand's population necessitates careful protection of whānau privacy and confidentiality. Therefore, the following anecdotal example is described in broad terms to minimize the chance of identifying individual whānau members. In this case, a hui was arranged to facilitate segregation testing within a large Māori whānau with a strong history of colorectal and endometrial cancer, in whom an *MLH1* variant of uncertain significance had been identified (Jackson et al. 2019). Following consultation with kaumātua, the hui was held at the whānau marae and involved a pōwhiri, mihi, and waiata, similar to the FH example described above. Fifteen whānau members spanning four generations attended, together with representatives from the New Zealand Familial Gastrointestinal Cancer Service and the Cancer Society. Subsequently, segregation data generated through this process were submitted to the international InSiGHT database as evidence supporting variant interpretation, and the genetic variant was soon after reclassified as pathogenic. This case highlights the value of culturally responsive approaches and of leveraging data held within clinical genetics services that care for ethnically distinct populations. Preparation for hui participation is supported by hospital training programs, as well as informal mechanisms including genetic counseling supervision and observation of more experienced colleagues.

**FIGURE 2:**
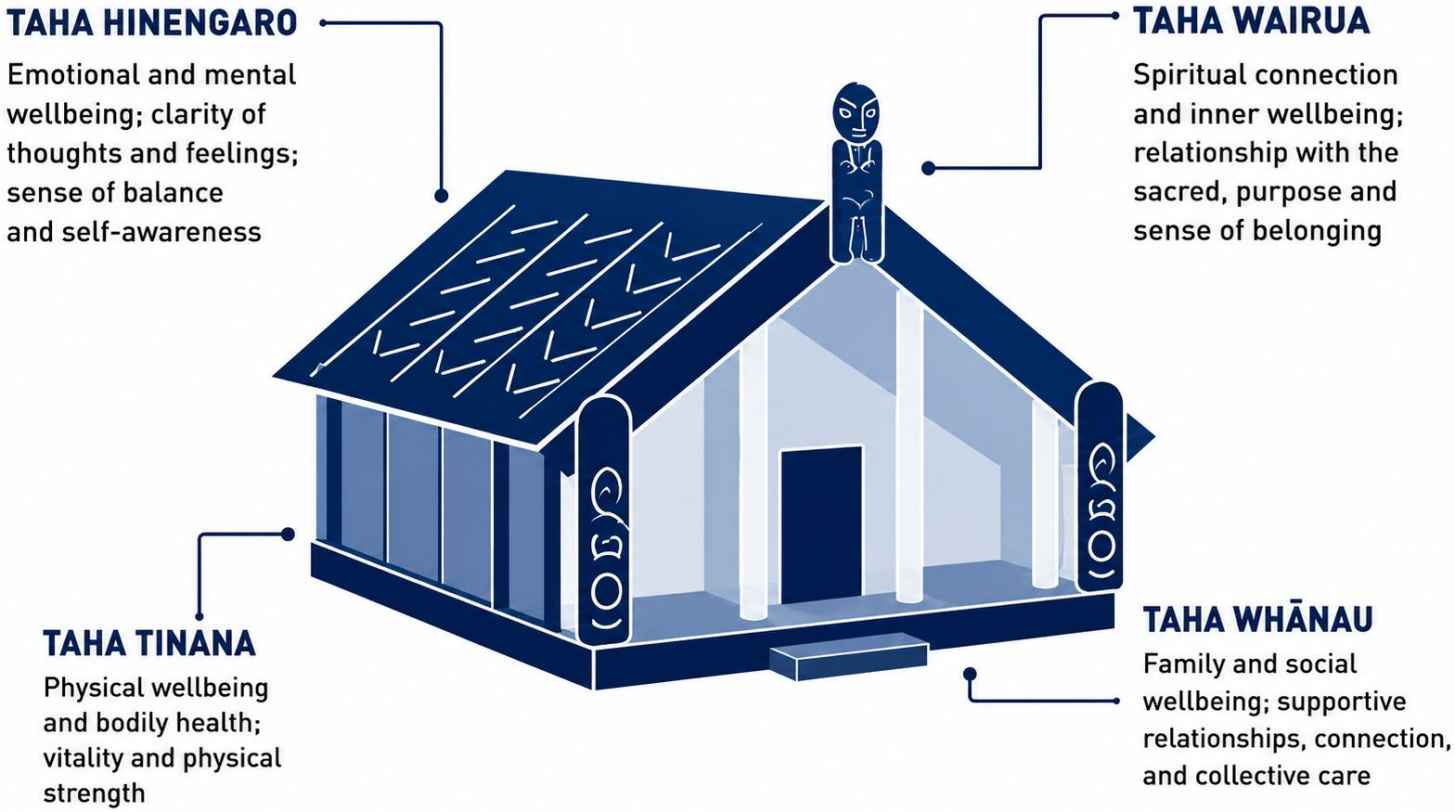
*Te Whare Tapa Whā—A Māori Model of Health*. This figure illustrates Te Whare Tapa Whā, a Māori model of health that conceptualizes wellbeing as a *wharenui* (meeting house) supported by four interconnected dimensions: Taha Tinana (physical health), Taha Hinengaro (mental and emotional health), Taha Wairua (spiritual health), and Taha Whānau (family and social health). This is a visual representation of the model described in “Whare Tapa Wha: A Māori Model of a Unified Theory of Health.” by T. Rochford 2004, *The Journal of Primary Prevention*. Image designed using Claude 4.6 Sonnet, Anthropic 2026, 14 May 2026.

Genetic counselors often use Māori models of health to inform holistic patient care. According to *Te Whare Tapa Whā* (Figure 2), there are four pillars of health: *Taha Tinana* (physical health), *Taha Wairua* (spiritual health), *Taha Whānau* (family health), and *Taha Hinengaro* (mental health). If one or more of these pillars is affected, an individual or group can become unbalanced and unwell. It is important to address each of these aspects of health in order to treat an illness effectively (Rochford 2004).

Building on the foundations of Te Whare Tapa Whā, other Māori health models, including *Te Wheke* and the *Meihana Model*, further describe the interconnected influences on health and wellbeing, encompassing concepts such as *mauri* (life force; vital essence), inherited strengths, and the central role of whānau (Pere and Nicholson 1991; Pitama et al. 2014). Together, these models provide a culturally informed framework for genetic counseling that considers genetic information within the context of whānau, wairua, *hinengaro* (mind; thoughts), and *tinana* (body; physical self). This holistic approach supports informed decision‐making, cultural safety, and *mana*‐enhancing (power; prestige) when working with Māori whānau (Curtis et al. 2019).

## 4 Partnership With Whānau

Māori‐led research is critical to ensuring healthcare in New Zealand is equitable and culturally safe. For Māori, genomic data sovereignty and governance are essential to rangatiratanga, informed decision‐making, and whānau/community wellbeing (Sporle et al. 2020). *Te Mana Raraunga*, the Māori Data Sovereignty Network, maintains that Māori data should be governed by Māori and that Māori have the right to decide how their data is collected and accessed (Kukutai et al. 2023). There is an increasing drive towards onshore genetic testing and data repatriation to maintain *kaitiakitanga* (guardianship). The *He Kākano* Aotearoa Variome project leads in this space, assembling a comprehensive database of genetic variation specific to Māori to improve the accuracy and inclusivity of genomic healthcare for this underrepresented population (Genomics Aotearoa 2024; Caron et al. 2020).

An early example of whānau‐initiated research led to the discovery of the *CDH1* tumor suppressor gene associated with hereditary diffuse gastric cancer (HDGC). In 1994, Maybelle McLeod sought to investigate the high incidence of gastric cancer within her whānau. Working in partnership with cancer genetic researchers from the University of Otago, marae‐based health services and affected whānau co‐led efforts that culminated in the identification of the first pathogenic variants in the *CDH1* gene (Guilford et al. 1998). As of 2025, 14 different pathogenic *CDH1* variants have been identified among Māori, with Māori accounting for an estimated 71%–77% of pathogenic variant carriers in New Zealand (Decourtye‐Espiard et al. 2025). Current HDGC practice guidelines developed by an advisory group, which included genetic counselors and HDGC advocates and whānau, recommend that all individuals of Māori ethnicity with a diagnosis of diffuse gastric cancer be offered genetic testing regardless of age (Blair et al. 2020). Working alongside HDGC advocates and whānau strengthens consensus on HDGC management and supports the development of recommendations that reflect the lived experiences of affected whānau. Genetic counselors have played a central role in initiating and maintaining long‐term partnerships with marae‐based health services, building trust, and supporting reciprocal education and early engagement with at‐risk whānau. This approach enables more timely referral to and uptake of clinical genetics services, strengthens continuity of care and follow‐up, and facilitates more effective and culturally responsive genetic counseling practice.

## 5 The Landscape of Genetic Services in New Zealand

Genetic counseling in New Zealand is delivered by a national publicly funded service, Genetic Health Service New Zealand (GHSNZ), which provides adult, pediatric, general, and cancer genetics care. As of 2026, 20 genetic counselors are employed by GHSNZ, equating to approximately 17 permanent full‐time equivalent (FTE) positions. GHSNZ operates through three regional hubs: Auckland (Northern), Wellington (Central), and Christchurch (South), each offering local and outreach counseling through face‐to‐face and telehealth appointments. Service delivery is determined by national eligibility criteria to maintain consistency across regions despite varying local resource availability. Although private practice represents a relatively small proportion of the genetic counseling workforce, it provides an alternative access pathway for individuals/families who do not meet public eligibility criteria or those who wish to avoid public wait times.

Three primary cytogenetics and molecular genetics laboratories currently serve the publicly funded health sector in New Zealand: LabPLUS (Auckland), Canterbury Health Laboratories (CHL; Christchurch), and Wellington Regional Genetics Laboratory. Each laboratory has progressively expanded its range of molecular tests; however, implementation is shaped by access to technology, international guidelines, and in response to the specific clinical needs of the New Zealand population. Current publicly available testing includes chromosomal microarray analysis, exome sequencing, hereditary cancer predisposition testing, other targeted multigene panels, and somatic tumor testing. Publicly funded Pre‐implantation Genetic Diagnosis is available via CHL for chromosomal and single‐gene conditions that meet criteria (The Advisory Committee on Assisted Reproductive Technology 2008). At present, no public laboratories employ genetic counselors to support genetic testing queries or oversee test utilization.

## 6 Genetic Counselor Training, Certification, and Regulation

The *Human Genetics Society of Australasia* (HGSA) is responsible for the training, certification, and professional regulation of genetic counselors in Australia and New Zealand. Genetic counselors are recognized as a distinct professional group (Hoskins et al. 2021), and genetic counselors who have completed a formal certification process can use the title *Fellow of the HGSA* (FHGSA). As New Zealand does not have a statutory body overseeing the regulation of health professionals (Hoskins et al. 2021), the regulation of the use of the FHGSA title falls under the Australian body *National Alliance of Self‐Regulating Health Professionals* (NASRHP). The title “genetic counselor” is not yet protected in New Zealand or Australia, meaning anybody can legally call themselves a genetic counselor, regardless of qualifications and clinical experience. Genetic counseling certification pathways underwent revision between 2010 and 2012, as the previous training pathway (a Graduate Diploma in Genetic Counseling) transitioned to a Master of Genetic Counseling (McEwen et al. 2013).

At present, there is no nationally accredited Genetic Counseling Masters program, requiring prospective students to undertake training overseas. Hybrid delivery options are available through the *University of Technology Sydney* (UTS); however, New Zealand citizens are not automatically eligible for Australian student loan programs, introducing significant financial barriers. These barriers disproportionately affect those from Māori, Pacific and lower socioeconomic backgrounds, limiting equitable access to entry into the profession. In response, UTS in partnership with *Pūtahi Manawa|Healthy Hearts for Aotearoa New Zealand* offered a scholarship for a Pasifika student in 2024 (Healthy Heart for Aotearoa New Zealand 2024).

Genetic counselors practicing in New Zealand operate with a comparatively high level of professional autonomy. In the majority of genetic counselor‐led cases, patients are managed throughout the testing process without involvement from a clinical geneticist, including responsibility for test selection and ordering. Clinical geneticists are involved in panel design; however, individual case input is typically reserved for complex patients, where multidisciplinary case review supports clinical decision‐making. In addition to direct patient care, genetic counselors in New Zealand play a central role in developing and maintaining national genetic testing guidelines.

## 7 Current Challenges: Workforce, Demand, and Service Delivery in New Zealand

The genetic counseling workforce in New Zealand faces a number of significant challenges. Ongoing workforce constraints have contributed to routine public wait times of approximately 10–15 months (Jackson et al. 2025). Consistent with this, genetic counselors working in the public system report a sustained increase in service demand (Kanga‐Parabia et al. 2024). GHSNZ has experienced a 20% annual increase in referrals (equating to an additional 1000 referrals per year) with no increase in permanent genetic counseling positions since 2016. On a per‐capita basis, workforce capacity in New Zealand remains markedly lower than in comparable countries, with approximately one genetic counselor per 315,000 people, compared with one per 59,000–74,000 in Australia and one per 210,000 in the United Kingdom (Jackson et al. 2025).

Increasing demand for genetic and genomic testing to inform diagnosis and clinical management has contributed to the expansion of mainstream genetic testing in New Zealand. Early adopters include neurologists, cardiologists, and pediatricians, who can order genetic testing for clinically affected patients under their care, supported by a multidisciplinary meeting (MDM) review where indicated. The extent of mainstream testing in these specialties remains variable across regions and providers. Treatment‐focused genetic testing in the oncology space has been more formally implemented to ensure national consistency, with structured onboarding and education for ordering clinicians. Mainstreaming for ovarian cancer began in Auckland in 2018, with a national rollout starting in 2022. In 2025, a national breast cancer mainstreaming pilot commenced, with plans to extend mainstream genetic testing to additional cancer types where results have treatment implications. If implemented successfully, these pathways could lead to a significant reduction in burden on clinical genetic services nationally and decrease wait times.

In New Zealand, both clinical and research bodies operate within a comparatively constrained funding environment. A lack of sustained investment in clinical services restricts workforce growth and service innovation, while limited research funding creates gaps in population‐specific data, resulting in an insufficient evidence base for policy development. In contrast, Australia's federal government has invested in genomics to a much greater extent, for example, introducing a publicly funded three‐condition reproductive genetic carrier screening (RGCS) panel for spinal muscular atrophy, cystic fibrosis, and fragile X syndrome (Medicare Benefits Schedule—Item 73,451). The HGSA recommends that RGCS be offered to all couples who are pregnant or planning a pregnancy (Vears et al. 2023). In New Zealand, however, no such screening program exists. The absence of equivalent investment has perpetuated reliance on privately funded offshore RGCS, restricting access to those with the financial means to self‐pay. For genetic counselors, this introduces the challenge of managing rising expectations of patients and clinicians seeking optimal care while practicing within a system where test availability lags behind that of neighboring countries, and access remains largely inequitable.

These constraints are further shaped by New Zealand's unique medicines funding landscape. New Zealand is the only country with a single national government agency, *Pharmaceutical Management Agency* (Pharmac), which is responsible for determining which medicines are funded and for managing the national pharmaceutical budget (Pharmac 2025). Decisions made by Pharmac directly influence access to germline genetic testing, as eligibility criteria are often tied to treatment availability. This has been particularly evident in oncology, for example, with the introduction of poly (ADP‐ribose) polymerase inhibitors (PARPi). The availability of PARPi has been a key driver of mainstream testing for patients with breast and ovarian cancer. Currently, the PARPi Olaparib is publicly funded only for women with ovarian cancer who carry a germline *BRCA1/BRCA2* pathogenic variant (Pharmac 2022).

The increasing volume and demand for genetic testing brings the issue of genetic discrimination to the forefront. In New Zealand, life and health insurers are currently permitted to use genetic results in the underwriting of policies (Shelling et al. 2022). This is at odds with practice in the United Kingdom and Australia, where voluntary moratoriums restrict such use, and in Canada, where legislative protections prohibit the use of genetic test results in insurance underwriting (Shelling et al. 2022). Evidence suggests that concern about potential insurance discrimination may discourage some individuals from pursuing clinically indicated genetic testing, with downstream implications for screening and disease prevention (Fraser et al. 2023). In response, a cross‐profession advocacy group, *Against Genomic Discrimination Aotearoa* (AGenDA), was formed in 2021 to raise awareness of genetic discrimination and to advocate for legislative reform prohibiting the use of genetic test information in insurance underwriting (Shelling et al. 2022).

## 8 Future Directions

Exponential advances in testing methodology, coupled with expanding applications and escalating demand, are rapidly reshaping healthcare. To keep pace with the challenges posed by the transition to an era of genomic medicine, New Zealand will need to move towards a cohesive, coordinated, system‐level reform (King 2024). Within this evolving landscape, genetic counselors represent a crucial intersection between patients, laboratories, research, and other healthcare specialists. Realizing the full potential of genomic medicine necessitates increased and sustained investment in the genetic counseling workforce, alongside adaptation to new models of service delivery, including mainstreamed genetic testing, interdisciplinary practice, and broader genomic education and capability‐building across the healthcare system. The profession must orient to the growing integration of artificial intelligence, ensuring these tools are implemented safely and ethically to support (not replace) patient‐centered, culturally responsive care. In 2023, GHSNZ submitted a formal report to the New Zealand Ministry of Health regarding the state of genetic health service delivery, with specific recommendations to increase patient access to care and improve efficiency (Genetic Health Service New Zealand Workstream Groups 2023). From this, a national genetic database to store, access, share, and link whānau genetic and whakapapa information was requested. Māori remain underrepresented across the workforce, limiting the system's ability to authentically reflect the population it serves. It is therefore imperative to support Māori/Pacific training pathways and retention to ensure genetic counseling in New Zealand upholds culturally safe care for whānau and meets its Tiriti obligations.

Genetic counselors in Aotearoa New Zealand enjoy the satisfaction of working within a publicly funded health system and the breadth of practice associated with a general service operating across multifarious cultural contexts. The approach taken to working with New Zealand's Indigenous Māori population, grounded in co‐design and partnership, provides an excellent example and model for other nations with similar population dynamics and equity issues. In this regard, New Zealand is a world leader. Despite these strengths, challenges remain in the provision of genetic counseling services, particularly in relation to workforce resourcing and continuous adaptation to the ever‐evolving genomic landscape.

## Author Contributions

**Sarah Collis:** writing – original draft, writing – review and editing. **Harry G. Fraser:** writing – original draft, writing – review and editing. **Kimberley Gamet:** writing – review and editing. **Alison McEwen:** writing – review and editing, conceptualization. **Alice Christian:** writing – review and editing. **Linda L. Cheng:** writing – original draft, writing – review and editing. **Elisha Swainson:** writing – original draft, writing – review and editing, project administration.

## Funding

The authors have nothing to report.

## Ethics Statement

The authors have nothing to report.

## Consent

The authors have nothing to report.

## Conflicts of Interest

The authors declare no conflicts of interest.

## Data Availability

The authors have nothing to report.
